# Correction: The center of pressure and ankle muscle co-contraction in response to anterior-posterior perturbations

**DOI:** 10.1371/journal.pone.0226093

**Published:** 2019-11-27

**Authors:** The PLOS ONE Staff

## Notice of Republication

This article was republished on November 8, 2019 to correct errors in the Author Contributions, typographical errors in the Introduction and Methods sections, and errors affecting Figs 1, 5, 6, and 7 that were introduced during the typesetting process. The publisher apologizes for the errors. Please download this article again to view the correct version. The originally published, uncorrected article and the republished, corrected articles are provided here for reference.

## Supporting information

S1 File Originally published, uncorrected article. (PDF)

S2 File Republished, corrected article. (PDF)
