# Supplementary material for: Correction: The center of pressure and ankle muscle co-contraction in response to anterior-posterior perturbations
Source: PLoS One. 2019 Nov 27;14(11):e0226093. doi: 10.1371/journal.pone.0226093 (PMC6880986; doi:10.1371/journal.pone.0226093)
Supplement: S1 File — (PDF) [file pone.0226093.s001.pdf]

PLOS ONE

RESEARCH ARTICLE

The center of pressure and ankle muscle co-contraction in response to anterior-posterior perturbations

Dongwon Kim[1,2]*, **Jong-Moon Hwang**[3,4]*

1 Department of Biongineering, School of Engineering, University of Maryland, College Park, MD, United States of America, **2** Department of Physical Therapy and Rehabilitation Science, School of Medicine, University of Maryland, Baltimore, MD, United States of America, **3** Department of Rehabilitation Medicine, Kyungpook National University Hospital, Daegu, Korea, **4** Department of Rehabilitation Medicine, School of Medicine, Kyungpook National University, Daegu, Korea

* dongwonkim@som.umaryland.edu (DK); hti82@knu.ac.kr (JMH)

Check for updates

Abstract

Though both contraction of agonist muscles and co-contraction of antagonistic muscle pairs across the ankle joint are essential to postural stability, they are perceived to operate independently of each other, In an antagonistic setup, agonist muscles contract generating moment about the joint, while antagonist muscles contract generating stiffness across the joint. While both work together in maintaining robustness in the face of external perturbations, contractions of agonist muscles and co-contractions of antagonistic muscle pairs across the ankle joint play different roles in responding to and adapting to external perturbations. To determine their respective roles, we exposed participants to repeated perturbations in both large and small magnitudes. The center of pressure (COP) and a co-contraction index (CCI) were used to quantify the activation of agonist muscles and antagonistic muscle pairs across the ankle joint. Our results found that participants generated moment of a large magnitude across the ankle joint—a large deviation in the COP curve—in response to perturbations of a large magnitude ($p<0.05$), whereas the same participants generated higher stiffness about the ankle—a larger value in CCI—in response to perturbations of a small magnitude ($p<0.05$). These results indicate that participants use different postural strategies pertaining to circumstances. Further, the moment across the ankle decreased with repetitions of the same perturbation ($p<0.05$), and CCI tended to remain unchanged even in response to a different perturbation following repetition of the same perturbation ($p<0.05$). These findings suggest that ankle muscle contraction and co-contraction play different roles in regaining and maintaining postural stability. This study demonstrates that ankle moment and stiffness are not correlated in response to external perturbations.

OPEN ACCESS

Citation: Kim D, Hwang J-M (2018) The center of pressure and ankle muscle co-contraction in response to anterior-posterior perturbations. PLoS ONE 13(11): e0207667. https://doi.org/10.1371/journal.pone.0207667

Editor: Andrea Macaluso, Universita degli Studi di Roma 'Foro Italico', ITALY

Received: July 17, 2018

Accepted: November 5, 2018

Published: November 29, 2018

Copyright: © 2018 Kim, Hwang. This is an open access article distributed under the terms of the Creative Commons Attribution License, which permits unrestricted use, distribution, and reproduction in any medium, provided the original author and source are credited.

Data Availability Statement: All relevant data are within the manuscript and its Supporting Information files.

Funding: The authors received no specific funding for this work.

Competing interests: The authors have declared that no competing interests exist.

1 Introduction

The center of pressure (COP) is a widely used indicator of neuromuscular control while standing [1–7]. The major role of COP is laid in maintaining whole-body balance by controlling the acceleration of the whole-body center of mass. COP is closely proportional to the moment about the ankle joint that is generated by the musculoskeletal system governing the ankle [1, 8, 9]. Meanwhile, it is questionable whether stiffness associated with the ankle joint can be predicted using COP curves alone while recovering balance after external perturbations.

A study in 2014 differentiated fallers from non-fallers using a mathematical model and concluded that a greater excursion in COP in response to an external translational perturbation results from a stiffer ankle joint [10]. However, while COP is closely related to the ankle moment, it would be thought to be oversimplified if ankle stiffness is estimated from COP curves alone, particularly in differentiating postural control with great stiffness from postural control without great stiffness. Ankle stiffness is predominantly related to muscle co-contractions, that is, simultaneous activities of antagonistic muscle pairs across the joint, when postural stability is threatened [11–13]. In a joint system under an antagonistic setup, the moment generated from the contraction of agonist muscles is not linearly related to the co-contractions of the antagonistic muscle pairs about the joint [8, 14–16].

Plantar flexion and dorsiflexion are achieved through contractions of muscles that govern the ankle joint including the tibialis anterior and gastrocnemius [11, 17]. Activation by the nervous system of these muscles leads to corrective actions to recover balance in response to postural threats. For example, the gastrocnemius muscle plays a major role in modulating COP during plantar flexion [9, 18]. When external perturbations are predictable, as well as when they are unpredictable, humans usually adopt anticipatory postural control strategies to recover balance, or attempt to avoid a loss of balance [2, 5, 19]. These anticipatory strategies often engage muscle contractions of the lower extremities during balance challenges. As participants become familiarized with perturbations, changes in muscle contractions would be expected [5, 11, 20–23].

Meanwhile, co-contraction of the ankle musculature alters the impedance of the musculoskeletal system against unexpected perturbations [24, 25]. Co-contraction could greatly increase the available time to counteract destabilizing forces [13]. As well, since muscle co-contraction is centrally controlled, experience-based adaptation appears in muscle co-contraction for motor responses [26, 27].

A number of studies have explored the relationship of ankle stiffness—co-contraction—to ankle moment—agonist muscle contraction—employing COP and/or center of gravity (COG) [28–30]. As a rule, these studies have been limited to evaluating postural stability in quiet stance. Also, no attempt has yet made to evaluate simultaneous changes in muscle contraction and co-contraction during adaptation to external perturbations. It remains unclear how each activity of antagonistic muscle pairs across the ankle joint are centrally modulated as the perturbation is familiarized. In this study, we strive to answer the question as to whether agonist muscle contractions and co-contractions of the antagonistic muscles around the ankle act to cope with an external threatening factor in a separate and distinct versus a synchronized manner.

Herein, we investigate what trends in the activities of antagonistic muscle pairs appear as they adapt to repeated perturbations. These efforts provide an insight into how the contraction and co-contraction of agonist and antagonist muscles work to regain postural stability with each role when it is compromised by perturbations. Our findings respond to the question posed in [10] regarding whether ankle stiffness can b estimated from COP curves alone. Moreover, we examine whether the learning effect resulting from repeated perturbations of

agonist and antagonist muscle pairs during contraction and co-contraction are retained by imposing the challenge of an unexpected perturbation. The activities of antagonistic muscle contractions about the ankle are quantified using COP, a force plate, and a co-contraction index with surface electromyography (EMG). Kinematic data in response to external perturbations are analyzed to examine whether they could be used as an indicator of contractions of the antagonistic muscles.

2 Methods

2.1 Participants

A total of 9 male volunteers, from Kyungpook National University, ranging in age from 25 to 37 years (age: 32.45 ±3.57 standard deviations (SD); height: 1.72 ±0.05 m; weight: 72.45 ±8.25 kg), were recruited for this study. All participants reported no histories of neurological or motor deficits. None of the participants had previous experience with perturbation experiments and were not made aware of our experimental design. All participants gave written informed consent to participant in the study. The experiment was approved by Kyungpook National University's Institutional Review Board. The participants' heights, weights, hip joint heights, and foot lengths were measured.

2.2 Apparatus

Fig 1 displays a schematic of the experimental setup and experimental data from this setup. The experimental setup was composed of a platform on which participants stood, an EMG measurement device, two miniature motion tracking devices, and two personal computers (PCs) running Windows 10 (Microsoft Corp. Redmond, WA, USA).

The platform was built with a force plate (EzForce Plate, i2A systems Co., Ltd., Daejeon, Korea) to be used for measuring COP on a linear guide rail (8 mm/revolution) that converted a brushed DC motor's (RE65, Maxon Motor, Sachseln, Switzerland) rotary motion into anterior-posterior translational motion. The perturbations that caused body sway in the sagittal plane were designed to be small, medium and large. While the small and medium perturbations were designed to produce a displacement of 2 and 4 cm for 0.3 s, respectively, the large perturbation generated a displacement of 6 cm for 0.3 s. The force plate was designed to immediately move back to the original position for 0.3 s. All perturbations drew sinusoidal motions. For example, displacement was designed as $6\mathbf{sin}(\pi/0.6t)$ cm in the case of the large perturbation. The design of these perturbations was believed to elicit a muscular and biomechanical response to the perturbations.

Surface EMG was recorded using an EMG system (LXM3201, LAXTHA Inc., Daejeon, Korea). EMG data were obtained from muscles associated with the ankle in each leg—the tibialis anterior (TA) and medial gastrocnemius (GAS) muscles, respectively. Prior to the electrode placement, the skin on the data collection sites was shaved, cleansed, and abraded by rubbing alcohol over the site. Disposable pre-gelled Ag-Agcl electrodes (Blue Sensor, Medicotest, Inc, Olstykke, Denmark) were placed parallel to the muscle fiber orientations a 2-cm interelectrode distance.

The two motion tracking units (MPU9250, InvenSense Inc., San Jose, USA) measured the acceleration values of the lower and upper body, respectively, in the anterior-posterior (a_{AP}), craniocaudal (a_{CC}) and lateral (a_L) directions. 3D printed cases were devised to include each of the tracking units to allow firm placement of these units on the participant's body aligned in the three directions. One unit was attached at 5% of the total height below the knee joint while the other unit was attached at 13% of the total height above the hip joint. Acceleration values were used to calculate the angles of the lower and upper body with regard to the vertical line,

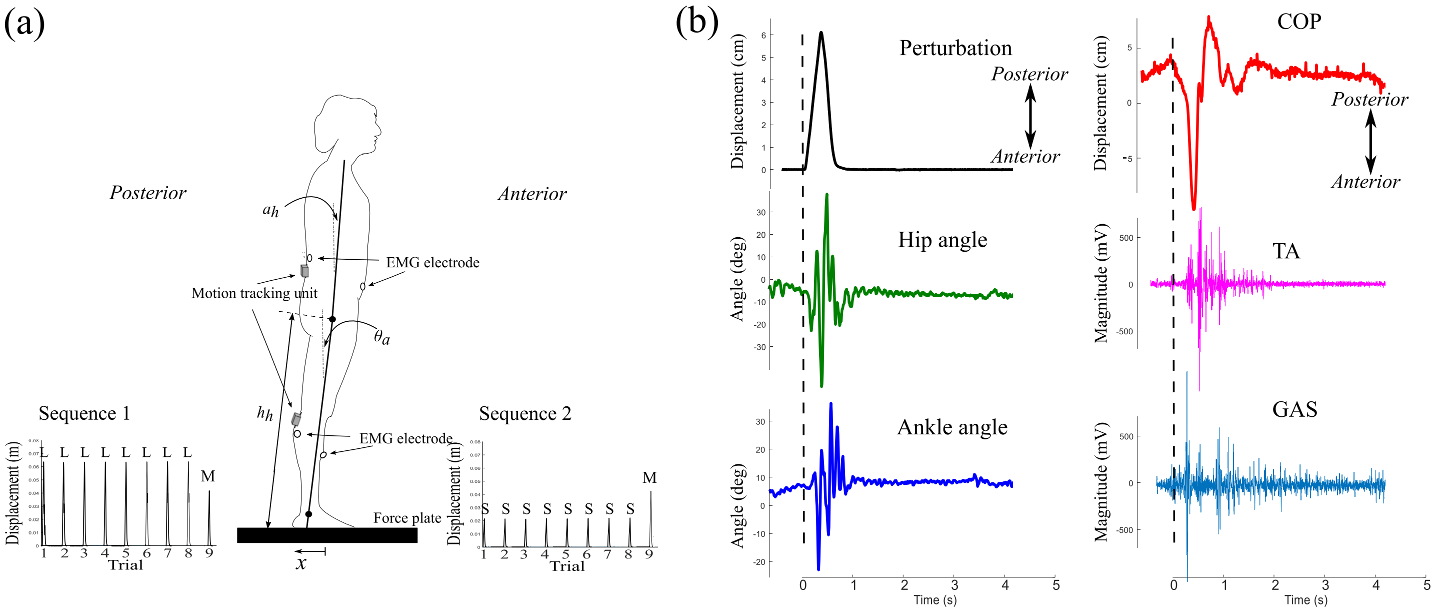

Fig 1. (a) A schematic of the experimental setup and definitions of the ankle angle θ_a, hip angle θ_h, and hip height h_h. Sequence 1 consists of 8 perturbations of a large magnitude followed by one perturbation of a medium magnitude. Sequence 2 consists of 8 perturbations of a small magnitude followed by one perturbation of a medium magnitude. (b) Experimental data obtained from a participant for Trial 2 in Sequence 1: displacement of the force plate, hip and ankle angles, COP, and EMG signals from the left leg. The dashed line indicates the time at the onset of the perturbation. All data are aligned to the dashed line.

https://doi.org/10.1371/journal.pone.0207667.g001

respectively, using the following equation:

$$\theta = \tan^{-1}\left(\frac{a_{AP}}{\sqrt{a_{CC}^2 + a_L^2}}\right). \tag{1}$$

Data including the DC motor angle, COP and body acceleration were acquired using a PC, and a control input was sent to the motor using a signal processing board (Batuino, i2A systems Co., Ltd., Daejeon, Korea), with a sampling frequency of 500 Hz. No filters were applied to the kinematic data to minimize signal distortion. The cables and interfaces were shielded to eliminate interference. EMG signals were acquired with a sampling frequency of 1024 Hz and were amplified with an overall gain of 1785 and digitized using Telescan 2.89 software (Laxtha, Daejeon, Korea). A band-pass filter (20–450 Hz) and a notch filter (60 Hz) were used. The data were then saved to another PC through serial communications. Signals were sent to both of the PCs for synchronization of the beginning and ending times of each experiment.

White noise was presented through headphones to eliminate the effect of noise from the apparatus on postural control.

2.3 Procedure

Throughout the experiment, participants stood barefoot on the force plate with their feet apart in a comfortable stance and their arms crossed over the chest. The participants were instructed to recover their balance every time a perturbation was administered and to avoid taking a step so long as they could avoid falling. They were also recommended to use the ankle strategy as far as possible, instead of the hip strategy or other strategies. The participants were not informed of the objective or design of the experiment.

The experiment consisted of a practice phase and a main phase. Before the main phase began, the practice phase was introduced to allow participants to familiarize themselves with the large and small perturbations. Participants were presented each perturbation twice.

In the main phase, all participants were evenly and randomly divided into two groups. Two sequences of perturbations were designed and administered in turn. Each group began with one of the two sequences. In one sequence, Sequence 1, the small perturbation was repeated 8 times, followed by one medium perturbation. In the other sequence, Sequence 2, the large perturbation was presented 8 times, followed by one medium perturbation. The perturbations were presented every 5 seconds. Participants were expected to regain and stabilize their original posture before the onset of the next perturbation. A break time of 5 minutes was provided between the two sequences in an effort to avoid fatigue and reduce the learning effect from the proceeding sequences.

2.4 Signal post-processing and Measures

All data analyses were completed using custom software (MATLAB vR2016b, Mathworks, Natick, MA). All acquired data were synchronized based on the onset of the perturbation during each trial using synchronization signals. COG was approximated using the following equation with the assumption that all joints other than the hip and ankle were locked, and that the center of mass was located near 13% of the total height away from the hip joint (defined as L_2) [1, 31]:

$$COG = L_1\theta_{Ankle} + L_2\theta_{Hip}, \tag{2}$$

where L_1 denotes the length between the hip joint and the bottom of the feet along the legs and θ_{Ankle}, θ_{Hip} denote the angles of the ankle and hip joints, respectively.

The levels of ankle angle, hip angle, COP, and COG were adjusted to 0 at the time when the perturbations began. To extract participants' postural strategies from these data, we considered how large the deviation from their original position was and in what ranges they moved in response to perturbations. Excursion I was defined as the distance between the maximum deviation in COP, and the kinematic data (ankle angle, hip angle and COG) resulting from the perturbation and their original position before the perturbation. Excursion II denoted the total anterior–posterior movement of COP and the kinematic data in response to the perturbation. Since COP is closely proportional to the ankle moment, Excursions I and II in COP could indicate how much ankle moment was generated to regain postural stability. Also, Excursions I and II in the kinematic data indicate the extent of body sway caused by the perturbations.

The stored EMG data were processed by subtracting the mean obtained from each trial, full wave rectifying and linear enveloping [30, 32]. Also, the data were further low-pass filtered using a second-ordered, zero-phase-lag Butterworth filter with a cutoff frequency of 4 Hz. Muscle activities in response to perturbations were believed to be at frequencies under 4 Hz. A co-contraction index (CCI) was adopted, which was proposed in [33, 34], to quantify the co-contraction effect between a pair of agonist and antagonist muscles over a specified time period. CCI is a common measure for analyzing both dynamic and static activities, and is expressed as follows:

$$\mathrm{CCI} \triangleq 2\sum_{i=1}^{N} \frac{min[\mathrm{EMG}_{GAS}(i), \mathrm{EMG}_{TA}(i)]}{\mathrm{EMG}_{GAS}(i) + \mathrm{EMG}_{TA}(i)} \times 100\%, \tag{3}$$

where *min* denotes the minimum value of the two elements.

Integer N is the total number of data points for the time window of interest, and EMG_{TA} and EMG_{GAS} denote the relative magnitudes of the linear enveloped EMG at each instant in

time for the muscle pairs under consideration. CCI was evaluated over 5 time periods of 100 ms ($N = 1024$) each: Period 1 was from -50 ms to 50 ms; Period 2 from 50 ms to 150 ms; Period 3 from 150 ms to 250 ms; Period 4 is from 250 ms to 350 ms; and Period 5 from 350 ms to 450 ms, based on the onset of the perturbations. The preset co-contraction in advance of the voluntary postural control actions [35] and the co-contractions occurring afterward could be investigated by evaluating CCI in each of the time periods. For the co-contraction associated with the ankle, the values of CCI obtained from each leg were averaged.

To investigate changes in contractions of the GAS and TA muscles as perturbations continue, mean absolute value (MAV) was employed [36]. The MAVs of the EMG linear envelopes over 0-350 ms were calculated for each trial, and ratios of MAV for each trial to that of the first trial were obtained.

2.5 Statistical analysis

A repeated-measures analysis of variance (ANOVA), with sequence and trial as the independent variables, was used to the evaluate performance changes across repeated measurements. If the sphericity assumption in ANOVAs was violated, then Greenhouse-Geisser adjusted p-values were used. Bonferroni post-hoc tests were conducted if a significant interaction effect was found. The statistical analyses were performed with SPSS version 20.0 (SPSS Inc., Chicago, USA) and the significance level was set at 0.05. All analyses were preceded by Shapiro–Wilk tests of normality and their results were employed only when normality was not violated. Measures during Trial 1 were excluded from analysis as they were regarded as a continuation of familiarization.

3 Results

For COP, as displayed in Fig 2, there were downward trends in the magnitudes of the first excursion (COP Excursion I) from Trial 1 to Trial 8. Participants generated greater excursions in COP during Sequence 1 than during Sequence 2. In Trial 9, COP Excursion I decreased from the same values in Trial 8 with a larger slope during Sequence 1 in comparison with Trial 8 from Trial 7, while COP Excursion I increased from Trial 8 to Trial 9 during Sequence 2.

For muscle co-contractions, downward trends were not observed for both sequences, as seen in Fig 3. No significant changes in CCI were identified as the trials continued. We observed that the values of CCI for participants in Sequence 2 were slightly greater than those for Sequence 1 during Period 2 in contrast to Period 1 during which voluntary responses did not work [35].

For muscle contractions, the activity of the medial GAS decreased as participants familiarized themselves with the repeated perturbations, while that of TA did not exhibit a downward trend, as shown in Fig 4.

The changes in the magnitudes of the ankle angle and COG exhibited downward trends from Trial 2 to Trial 8 (Fig 5). The magnitude of hip angle showed plateaus during this period.

3.1 Adaptation to repeated perturbations (Trial 2 to Trial 8)

Repeated-measures ANOVAs were performed on each measure from Trial 2 to Trial 8 with trial (seven levels: Trials 2–8) and sequence (two levels: Sequences 1 and 2) as within-subjects variables. **COP**: ANOVA on Excursion I of COP showed a significant main effect of sequence [$F(1, 8) = 43.796,\ p < 0.001,\ \eta_p^2 = 0.294$] and a significant main effect of trial [$F(6, 48) = 4.796,\ p = 0.001,\ \eta_p^2 = 0.373$]. The interaction effect was not significant ($p > 0.1$). ANOVA on Excursion II of COP produced a significant main effect of sequence

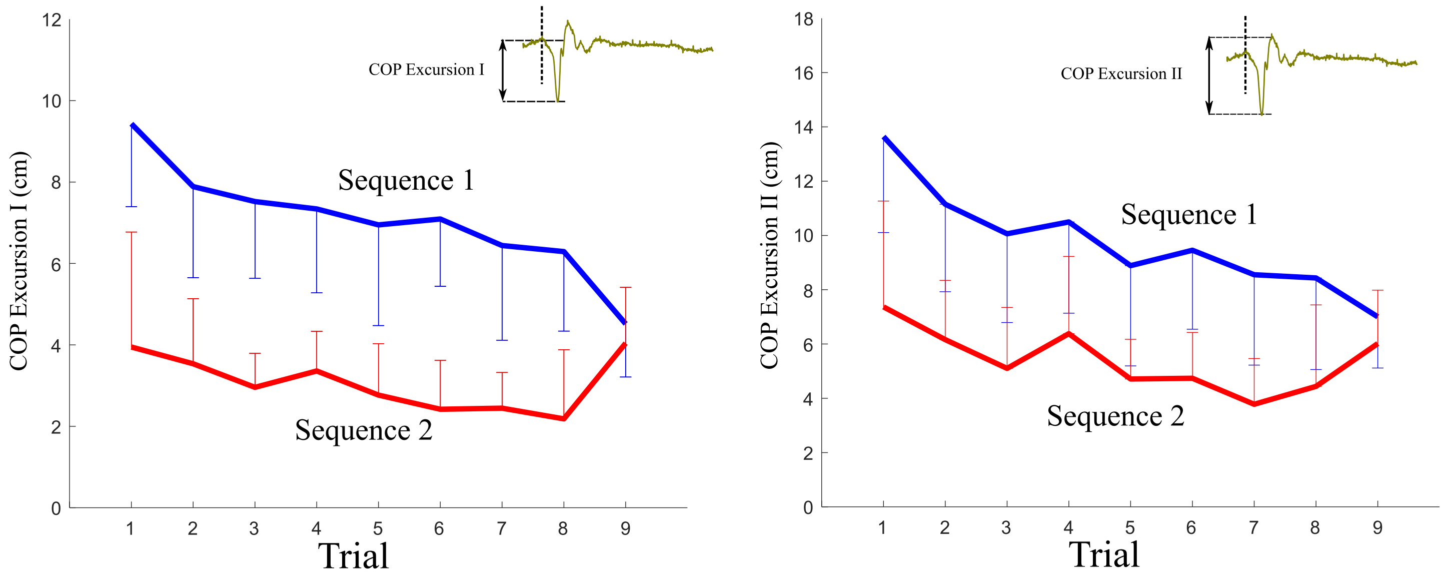

Fig 2. Mean by sequence of the magnitudes of Excursion I and Excursion II of COP for Trials 1-9 (Error bars are ± 1 standard deviation of the mean). The inlets display the definitions of COP Excursion I and Excursion II.

https://doi.org/10.1371/journal.pone.0207667.g002

$[F(1,8) = 15.119,\ p = 0.005,\ \eta_p^2 = 0.654]$ and a significant main effect of trial $[F(6,48) = 6.595,\ p < 0.001,\ \eta_p^2 = 0.452]$. **Co-contraction**: For Period 2, a marginally significant main effect of sequence $[F(1,8) = 5.078,\ p = 0.054,\ \eta_p^2 = 0.388]$ was observed. No other effects were found to be significant. No significant main effects and interactions were reported for Periods 1, 3, 4, and 5 (all $p > 0.1$). **Contraction**: ANOVA on MAV of GAS reported a marginally significant main effect of sequence $[F(1,7) = 4.720,\ p = 0.066,\ \eta_p^2 = 0.403]$ and a significant main effect of trial $[F(6,42) = 2.448,\ p = 0.04,\ \eta_p^2 = 0.259]$. One of the participants was excluded from the analysis as the contraction ratios between Trial 2 and Trial 8 were far greater than 3 standard deviations above the mean. Meanwhile, ANOVA on MAV of TA reported no main effects (all $p > 0.1$).

ANKLE: ANOVA on Excursion I of the ankle angle reported a significant main effect of sequence $[F(1,8) = 32.981,\ p < 0.001,\ \eta_p^2 = 0.805]$ and a significant main effect of trial

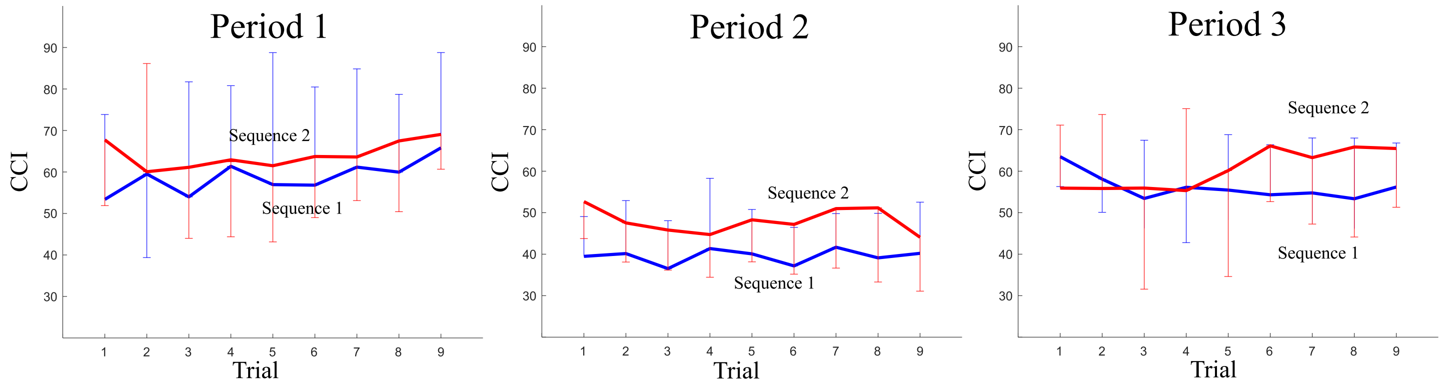

Fig 3. Mean by sequence of CCIs within each period: Period 1 (± 50 ms from the onset of perturbations), Period 2 (between 50 ms and 150 ms from the onset of perturbations) and Period 3 (between 150 ms and 250 ms from the onset of perturbations) for Trials 1-9 (Error bars are ± 1 standard deviation of the mean).

https://doi.org/10.1371/journal.pone.0207667.g003

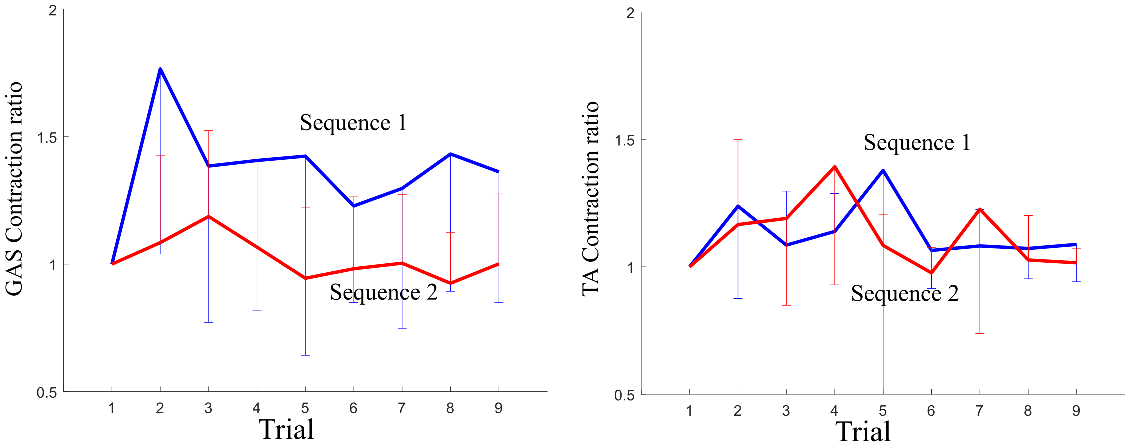

Fig 4. Mean by sequence of the ratios of the MAV (mean absolute value) of each trial to that of the first trial for Trials 1-9 (Error bars are ± 1 standard deviation of the mean).

https://doi.org/10.1371/journal.pone.0207667.g004

$[F(6, 48) = 3.417,\ p = 0.007,\ \eta_p^2 = 0.299]$. Meanwhile, ANOVA on Excursion II showed a significant main effect of sequence $[F(1, 8) = 47.658,\ p < 0.001,\ \eta_p^2 = 0.851]$. No significant main effect of trial was identified (p >0.4). **HIP**: ANOVA on Excursion I of the hip angle reported a significant main effect of sequence $[F(1, 8) = 91.155,\ p < 0.001,\ \eta_p^2 = 0.919]$. No significant main effect of trial was observed (p >0.7). Meanwhile, ANOVA on Excursion II displayed a significant main effect of sequence $[F(1, 8) = 72.572,\ p < 0.001,\ \eta_p^2 = 0.901]$. No significant main effect of trial was reported (p >0.5). **COG**: ANOVA on Excursion I of COG produced a significant main effect of sequence $[F(1, 8) = 44.310,\ p < 0.001,\ \eta_p^2 = 1.057]$ and a significant main effect of trial $[F(6, 48) = 3.964,\ p = 0.003,\ \eta_p^2 = 0.331]$. ANOVA on Excursion II of COG showed a significant main effect of sequence $[F(1, 8) = 47.854,\ p < 0.001,\ \eta_p^2 = 0.857]$. No significant main effect of trial was identified (p >0.2).

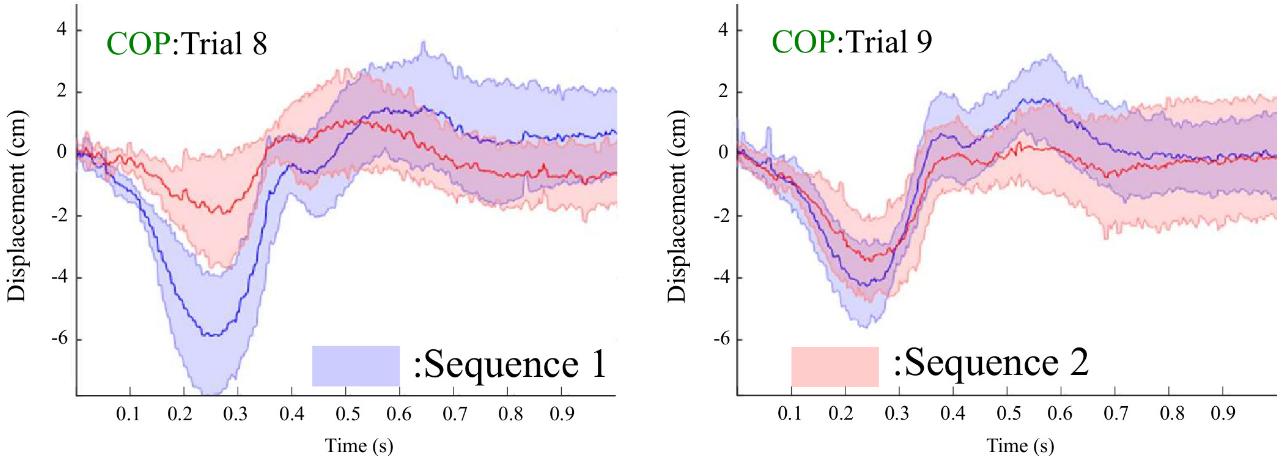

Fig 5. Mean by sequence of the magnitudes of Excursion II of (a) ankle angle, (b) hip angle and (c) COG for Trials 1-9 (Error bars are ± 1 standard deviation of the mean). The inlets show the definitions of Excursion I and Excursion II.

https://doi.org/10.1371/journal.pone.0207667.g005

3.2 Response to unexpected perturbations (Trial 8 to Trial 9)

Trial 8 was the last trial in the sequence of perturbations of large and small magnitudes. We compared the measures of Trial 8 with those of Trial 9 during which a perturbation of medium magnitude was given. Repeated-measures ANOVAs were conducted on each measure from Trial 8 to Trial 9 with trial (two levels: Trials 8 and 9) and sequence (two levels: Sequences 1 and 2) as within-subjects variables.

COP: ANOVA on Excursion I of COP showed a significant main effect of sequence $[F(1,8) = 14.759,\ p = 0.005,\ \eta_p^2 = 0.684]$ and a significant interaction $[F(1,8) = 12.226,\ p = 0.008,\ \eta_p^2 = 0.604]$. A Bonferroni post-hoc test showed that there was a significant difference between Sequences 1 and 2 for Trial 8 ($p = 0.003$); however, no significant difference was found for Trial 9 ($p > 0.4$). The main effect of trial was not significant ($p > 0.8$). The results on Excursion II of COP reported a significant main effect of sequence $[F(1,8) = 6.013,\ p = 0.040,\ \eta_p^2 = 0.429]$. The main effect of trial was not significant ($p > 0.05$). Fig 6 displays the means and ± 1 standard deviations of COP traces for 1 second after the onset of each perturbation across Trials 8 and 9. **Co-contraction**: For Period 3, as shown in Fig 7, ANOVA on CCI reported a significant main effect of sequence $[F(1,8) = 5.624,\ p = 0.045,\ \eta_p^2 = 0.413]$. The main effect of trial was not found to be significant ($p > 0.5$). Further, no main effects or interactions were identified for Periods 1, 2, 4, and 5 (all $p > 0.1$). **Contraction**: ANOVA on the contraction ratio for GAS reported a significant main effect of sequence $[F(1,7) = 5.962,\ p = 0.033,\ \eta_p^2 = 0.499]$. No other significant effects were reported for the GAS and TA contraction ratios.

Fig 8 displays tracings of ankle angle, hip angle and COG for 1 second after the onset of each perturbation in Trials 8 and 9. **ANKLE**: The results on Excursion I of the ankle angle reported a significant main effect of sequence $[F(1,8) = 16.457,\ p = 0.004,\ \eta_p^2 = 0.673]$ and

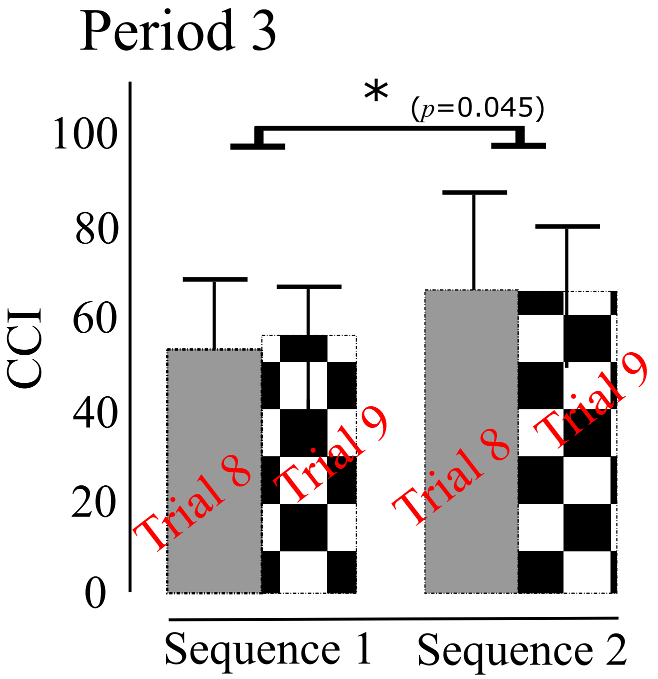

Fig 6. Means (bold) and ± 1 standard deviations (translucent) of COP traces for 1 second after the onset of each perturbation across Trial 8 and Trial 9. Blue: Sequence 1, Red: Sequence 2.

https://doi.org/10.1371/journal.pone.0207667.g006

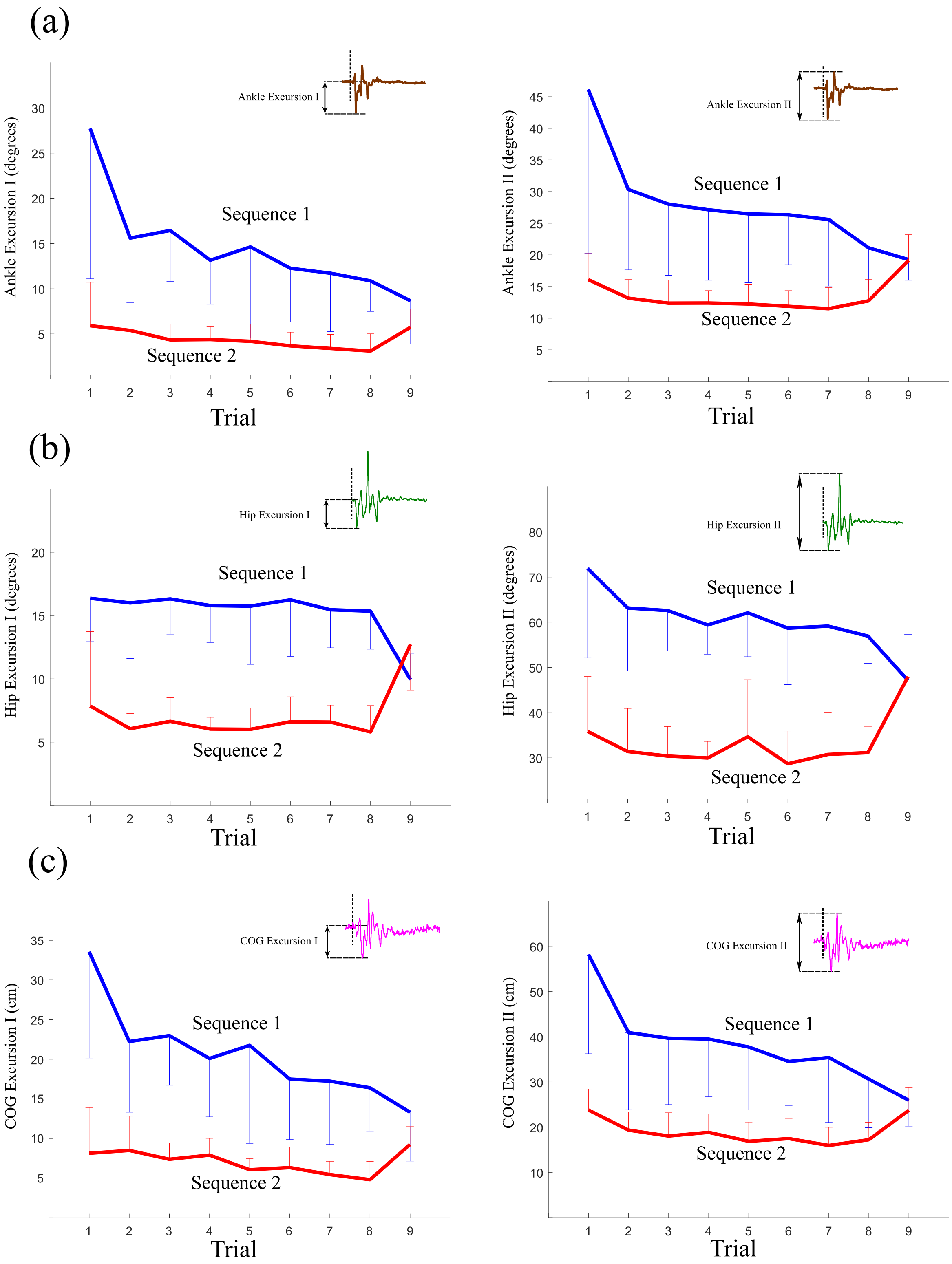

Fig 7. A comparison in the mean of CCI between Sequence 1 and Sequence 2 across Trials 8 and 9 for Period 3. There is no significant main effect of trial (ANOVA, $p > 0.5$), while a significant main effect of sequence is exhibited (ANOVA, $p = 0.045$). Error bars are ± 1 standard deviation of the mean. An asterisk indicates a significant main effect of sequence.

https://doi.org/10.1371/journal.pone.0207667.g007

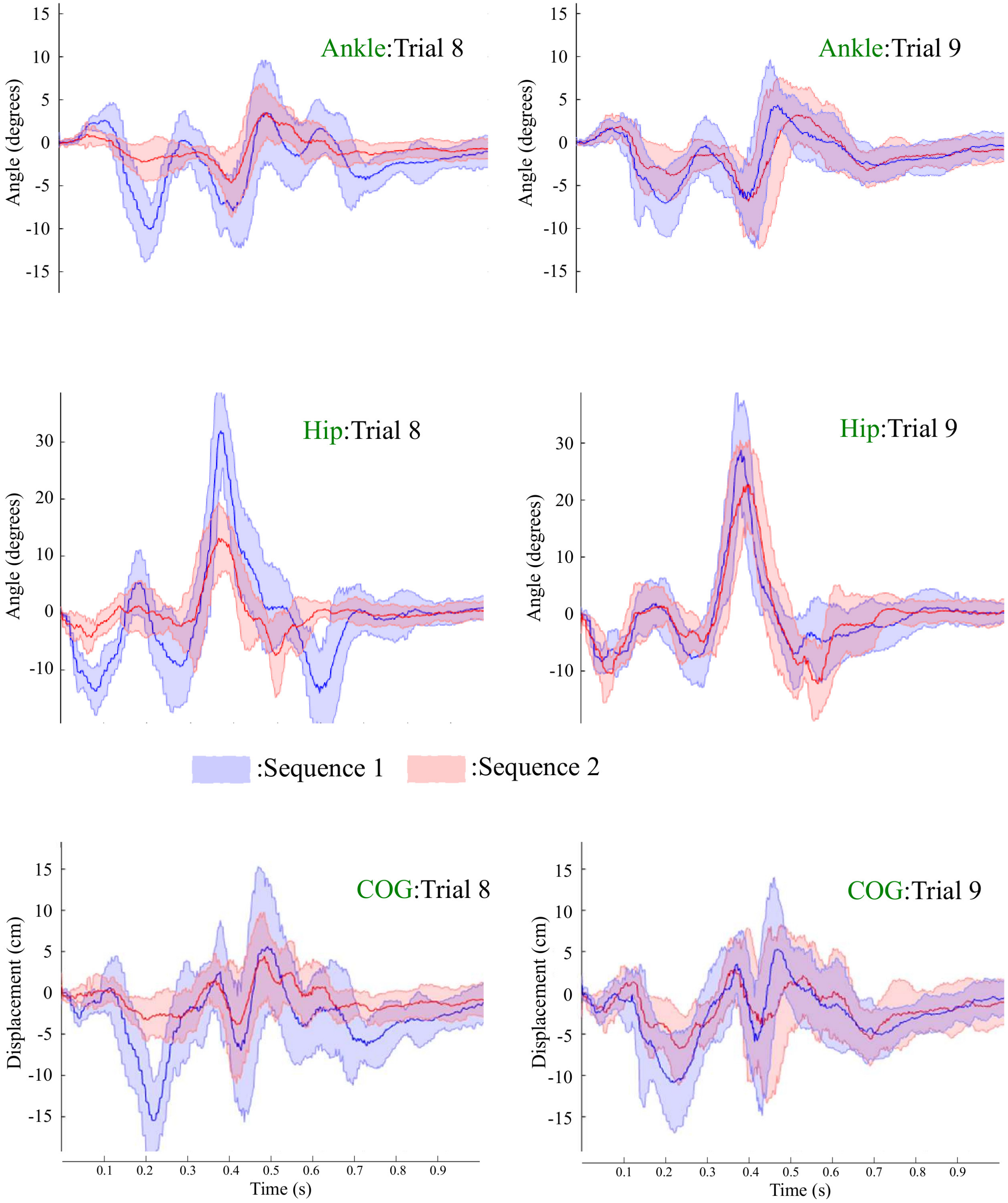

Fig 8. Means (bold) and ± 1 standard deviations (translucent) of traces of ankle angle, hip angle and COG for 1 second after the onset of each perturbation across Trial 8 and Trial 9. Blue: Sequence 1, Red: Sequence 2.

https://doi.org/10.1371/journal.pone.0207667.g008

a significant interaction effect $[F(1, 8) = 7.259,\ p = 0.027,\ \eta_p^2 = 0.476]$. A Bonferroni post-hoc test produced a significant difference between Sequences 1 and 2 for Trial 8 ($p = 0.001$) while no significant difference was reported for Trial 9 ($p = 0.129$). The main effect of trial was not significant ($p > 0.5$). The results on Excursion II reported a significant main effect of sequence $[F(1, 8) = 6.510,\ p = 0.034,\ \eta_p^2 = 0.449]$ and a significant interaction effect $[F(1, 8) = 12.206,\ p = 0.008,\ \eta_p^2 = 0.604]$. A Bonferroni post-hoc test produced a significant difference between Sequences 1 and 2 for Trial 8 ($p = 0.007$), whereas only a marginally significant difference was reported for Trial 9 ($p = 0.094$). The main effect of trial was not significant ($p > 0.1$). **HIP**: The results of Excursion I of the hip angle showed a significant main effect of sequence $[F(1, 8) = 18.134,\ p = 0.003,\ \eta_p^2 = 0.694]$. ANOVA also showed a significant interaction effect $[F(1, 8) = 211.034,\ p < 0.001,\ \eta_p^2 = 0.963]$. A Bonferroni post-hoc test produced a significant difference between Sequences 1 and 2 for Trial 8 ($p < 0.001$) and Trial 9 ($p = 0.014$). The results on Excursion II reported a significant main effect of sequence $[F(1, 8) = 10.609,\ p = 0.012,\ \eta_p^2 = 0.570]$ and a significant interaction effect $[F(1, 8) = 110.836,\ p < 0.001,\ \eta_p^2 = 0.933]$. A marginally significant effect of trial $[F(1, 8) = 5.125,\ p = 0.053,\ \eta_p^2 = 0.390]$ was reported. A Bonferroni post-hoc test produced a significant difference between Sequences 1 and 2 for Trial 8 ($p < 0.001$), whereas no significant difference was observed for Trial 9 ($p = 0.859$). **COG**: The results on the first excursion of COG produced a significant main effect of sequence $[F(1, 8) = 17.523,\ p = 0.003,\ \eta_p^2 = 0.687]$ and a significant interaction effect $[F(1, 8) = 10.343,\ p = 0.012,\ \eta_p^2 = 0.564]$. A Bonferroni post-hoc test produced a significant difference between Sequences 1 and 2 for Trial 8 ($p = 0.001$) while a marginally significant difference was reported for Trial 9 ($p = 0.097$). The main effect of trial was not significant ($p > 0.5$). The analysis of Excursion II for COG showed a significant main effect of sequence $[F(1, 8) = 8.836,\ p = 0.018,\ \eta_p^2 = 0.525]$ and a significant interaction effect $[F(1, 8) = 8.266,\ p = 0.021,\ \eta_p^2 = 0.508]$. A Bonferroni post-hoc test produced a significant difference between Sequences 1 and 2 for Trial 8 ($p = 0.009$), while no significant difference was reported for Trial 9 ($p > 0.3$). The main effect of trial was not significant ($p > 0.6$).

4 Discussion

This study was originally motivated by the question as to whether COP could be an indicator of ankle muscle co-contraction, with the assumption that ankle stiffness is predominantly related to muscle co-contractions in a situation where external perturbations threatens postural stability [13]. This question is closely tied to the relationship between simultaneous contractions in antagonistic muscle pairs during balance recovery. As the human's mechanical power is certainly limited, it would be difficult to increase the contraction of the agonist muscles and co-contraction of the pairs at the same time. Rather, contraction of agonist muscles or co-contraction of antagonistic muscle pairs would be selected and emphasized according to the perturbed circumstances. Also, it could be that contractions and co-contractions exhibit a different trend of adaptation in response to repeated perturbations. The results presented in this paper provide an insight into the roles of muscle contraction and co-contraction in terms of COP and CCI. In addition, we investigated the behaviors of ankle angle, hip angle and COG, which are the results of muscle contraction and co-contraction.

4.1 Adaptation to repeated perturbations (Trial 2 to Trial 8)

Overall, the magnitude of the excursion in COP curves decreased as the perturbations of the same magnitudes were repeated ($p < 0.05$), as shown in Fig 2. This indicates that muscle

contractions generating the counteracting moment to leaning continued to be tailored to external perturbations. In particular, the magnitudes of the EMG envelope of GAS showed a downward trend between Trial 2 and Trial 8 ($p < 0.05$), which is in accordance with the trend of COP. This may be because plantar flexion was dominant over dorsiflexion in our perturbation design (in particular, COP Excursion I reflected this fact). The GAS muscle played a major role in modulating COP in comparison with the TA muscle in our study [9, 18]. There is clear evidence that central activation does change because the EMG activities of the ankle muscles are modulated in anticipation of postural sway [2, 11], e.g.,]. Our results agree with the evidence.

For CCI, participants displayed a flat trend for all the considered time periods across all trials. Thus, they maintained a similar level of ankle muscle co-contraction (see Fig 3). Muscle co-contraction would be expected to vary with adaptation to external perturbations [8, 37, 38]; however, this was not the case. From the results showing that COP and CCI follow different trends, we could postulate that muscle contractions in GAS and TA were activated in a harmonious manner. Not only was GAS excited to counteract the perturbations, but also TA was excited to modulate ankle stiffness during balance recovery. One study proposed that the activities of the medial GAS and TA motor units are synchronized in a short-term manner [39]. A common synaptic input to the GAS and TA motor neurons could serve to simultaneously activate these two muscles during co-contraction. Our findings indicating that co-contraction remains the same may support the existence of the common input, although the activity of TA did not exhibit a downward trend in our analysis.

Participants who experienced Sequence 1, consisting of perturbations of large magnitude, generated a large range of COP behaviors in the anterior-posterior direction, in comparison to when they experienced a train of perturbations of small magnitude (Sequence 2). This implies that more muscle contraction was activated to generate a moment at the ankle joint in response to the perturbations during Sequence 1 in comparison to that during Sequence 2. Meanwhile, Sequence 2 produced a marginally significantly greater CCI than Sequence 1 for Period 2, about which the voluntary movement began (50 ms after the perturbation onset, $p = 0.054$). It would be possible to interpret the results as participants tended to employ greater co-contraction to minimize body sway caused by a perturbation of small magnitude, rather than emphasizing that GAS contraction recovered the leaned body in response to a perturbation of large magnitude. An increase in co-contraction typically occurs to minimize postural sway [40–42].

Note that participants did not prepare a postural strategy in advance of the perturbations. Ankle muscle co-contraction was not modulated for Period 1 (see Fig 3). The extent of ankle muscle co-contraction did not vary significantly with the trials or the magnitude of perturbations before the onset of voluntary reaction to postural threats.

The extents of the excursions in COG as well as in ankle angle decreased as the trials advanced to Trial 8 ($p < 0.05$). This similar trend to COP would be associated with the flat trend of CCI. Stiffness can be regarded as the ratio between the angular displacement and moment around the ankle joint [43]. Since COG reflects the ankle's angular displacement, to say nothing of ankle angle, while COP reflects the ankle moment, it would be possible to speculate that the flat trend of CCI led to similar trends between COP and COG. Typically, perturbations of large magnitude resulted in larger excursions in comparison with perturbations of small magnitude. The smaller excursions may have also resulted from greater co-contraction of the antagonistic muscles during Period 2. Intuitively, a 1 degree-of-freedom inverted pendulum with high joint stiffness shows the smaller magnitude of sway in response to an external force, in comparison to one with low joint stiffness, [1]. This phenomenon will be addressed again.

4.2 Response to unexpected perturbations (Trial 8 to Trial 9)

During Trial 8, the learning effect on a certain perturbation could be considered the maximum in this experimental design, while at Trial 9, a perturbation of a different magnitude was introduced to participants. We zoomed in on the differences in the measures of interest during Trials 8 and 9. For COP, we observed that there was no significant difference in the extents of the first excursion between Sequences 1 and 2 during Trial 9 in contrast to Trial 8 (see Fig 6). Meanwhile, for Excursion II, the ANOVA results showed that participants in Sequence I produced greater excursions than in Sequence II during the two trials.

The statistical results on the contraction ratios of GAS were not the same as those on COP during Trials 8 and Trial 9, in contrast to the time period from Trials 2 to 8. Since the contraction ratios were calculated based on muscle activity at Trial 1, it might not be reasonable to interpret these results by directly comparing the contraction ratios between Sequences 1 and 2 in these results.

With respect to ankle stiffness evaluated with CCI, we found that participants adopted a greater stiffness in Sequence 2 than Sequence 1 for Period 3. Note that participants maintained a similar extent of ankle stiffness across the two trials, regardless of the sequence. These results demonstrate that co-contractions might be influenced by the previous trial in an anticipatory manner. The results are similar to those showing that the level of CCI did not change across trials from Trial 2 to Trial 8.

It appears that a higher CCI led to smaller excursions in ankle angle and COG during Sequence 2 in comparison with that during Sequence 1, though the *p*-value failed to reach the significance level in Trial 9. Also, the significant difference in COP Excursion II between the two sequences could be explained by the difference in ankle angle or COG. Participants in Sequence 2 generated ankle moments of smaller magnitude than in Sequence 1 to counteract body sway, for which the magnitude was decreased by higher stiffness for Period 3.

4.3 General remarks

The contraction of agonist muscles as well as the co-contraction of antagonistic muscle pairs is employed by the CNS in voluntary motor tasks. Anticipatory strategies often govern muscle contraction and co-contraction of the lower extremities when postural stability is threatened. Ankle muscle contractions produce counteracting forces through plantar flexion and dorsiflexion in response to balance challenges [9, 11, 17]. Muscle activities at the ankle joint correlate with the behavior of COP [9, 18], which also correlates with the ankle moment [1, 8, 9].

Muscle co-contraction regulates the impedance of the musculoskeletal system, minimizing body sway from internal or external perturbations [24, 25, 40–42]. Increasing joint stiffness through co-contraction of sufficient magnitude could completely counteract destabilizing perturbations. This phenomenon is also exhibited in upper-limb movements perturbed by unstable loads [44, 45]. Though the mechanical properties of the human ankle are modulated through feed-forward components including muscle co-contraction as well as feedback components including stretch reflexes, a reduced influence of feedback control during postural challenges has been reported [46–48]. The increased ankle stiffness during co-contraction is contributed primarily by the non-reflex components including activations of TA, medial GAS and peroneus longus muscles [13].

In this study, simultaneous behaviors in the contraction and co-contraction of antagonistic muscles responding to postural challenges are our main interest. We found that the extent of co-contraction of agonist and antagonist muscles about the ankle joint remained unchanged as participants adapt to perturbations, whereas the extent of contraction of agonist muscles decreased. These findings primarily indicate that it might not be reasonable to use COP curves

alone to estimate ankle stiffness, at least during perturbations that threaten balance. The ankle moment, which determines the behavior of COP, is generated by contracting the agonist muscles across the ankle joint, whereas ankle stiffness during voluntary movements largely involves the activities of the agonist and antagonist muscles. This fact suggests the irrationality of predicting the co-contraction of antagonistic muscle pairs using the contraction of the agonist muscles.

In our study, we observed that participants tended to be stiffer in response to perturbations of small magnitude. In contrast, they were less stiff and generated greater ankle moments against perturbations of large magnitude. Increasing muscle co-contraction is beneficial for minimizing body sway [40–42], but it also hinders the generation of a moment that is great enough to counteract balance challenges. The nervous system may perform a selective activation of the muscles across the ankle [13]. We emphasize that co-contraction showed a significant change in Period 3, which suggests that the CNS select its postural strategy after experiencing the ongoing perturbation, not prior to its onset.

Participants maintained a similar level of muscle co-contraction even when the magnitude of the given perturbation changed unexpectedly. This phenomenon would also imply that participants might have been intentionally selective of the stiffness level based on perturbations they experienced, regardless of how familiar they were with the perturbations. This finding could be related to the fact that people who have a fear of falling, or have experienced a fall in the past, tend to employ increased co-contractions in response to perturbations [42, 49, 50].

Though participants were encouraged to use the ankle strategy, hip angular displacements were noticeable (see Fig 8). These results may be interpreted by a mixed hip and ankle strategy that can be adopted to correct postural disturbances of any speed in the anterior-posterior direction, instead of using a pure ankle strategy, when the main objective of optimization is a minimal neural effort [51]. In our experiment, the ankle and hip angular displacements showed similar trends in excursions in response to repeated perturbations and an unexpected perturbation. This would mean that participants use the ankle strategy or mixed strategy consistently. Those strategies presumably produce a compensatory moment on the ankle joint to regain balance by moving the whole-body COM forward or backward. Here, we would like to emphasize that the main objective is originally to investigate the relationship between COP (ankle moment) and ankle muscle co-contraction, regardless of whether participants adopt the ankle strategy or other strategies.

The theories proposed in the study [10] are in contrast to those supported by the majority of studies in several aspects. A representative one is that the authors in the study, and through personal communication, denied the existence of feed-forward control in recovering perturbed balance, focusing on only feedback control involving transmission delays in feedback loops. In comparison with the arm, it is possible that stretch reflexes at the ankle have a limited contribution, because of longer transmission delays, slower muscle activation time constants and the high compliance of the Achilles tendon [13]. Nonetheless, the authors ignored the possibility of the existence of pre-programmed motor command or the involvement of ballistic movements that are generated based on an estimate by forward models of the body and the environment [52–54]. However, in their study ankle stiffness was linked to a fear of falling, a factor of feed-forward control based on experience, followed by their assertion that a fear of falling does not necessarily result in high stiffness. Although their study may be grounded in the equilibrium-point hypothesis [55], the transmission delay in the feedback loop they identified through the mathematical model is unrealistically short.

Another opposing theory presented in their study, which motivated our study, is that the authors estimated ankle stiffness in response to translational perturbations using ankle moment alone, which was calculated from COP. Using optimization, the output of the model

was fitted with the empirical COP data. However, fitting to the COP curves only (without an angular displacement component) would promote an insensitivity to estimated ankle stiffness. It is possible that the optimization provided a wide range of estimated stiffness, even within similar levels of the goodness of fit. The authors suggested that the non-faller group employed greater stiffness, which would imply that the group showed smaller magnitudes of body sway [40–42]. This suggestion contradicts their COG data. Though the study population was composed of pregnant women in their study, the authors basically investigated the participants' neuromuscular control based on COP curves alone.

Supporting information

S1 File. COP. The COP data are included.
(XLSX)

S2 File. Ankleangle. The angle data are included.
(XLSX)

S3 File. Hipangle. The hip data are included.
(XLSX)

S4 File. EMGsignals. The EMG data are included.
(MAT)

Acknowledgments

We would like to express our heart-felt gratitude to i2A Systems, Co., Ltd. for their efforts in building the experimental apparatus.

Author Contributions

Conceptualization: Dongwon Kim.

Data curation: Dongwon Kim.

Formal analysis: Dongwon Kim.

Funding acquisition: Jong-Moon Hwang.

Investigation: Dongwon Kim.

Project administration: Jong-Moon Hwang.

Supervision: Dongwon Kim.

Validation: Dongwon Kim.

Visualization: Dongwon Kim.

Writing – original draft: Dongwon Kim.

References

1. Winter DA. ABC (anatomy, biomechanics and control) of balance during standing and walking. Waterloo Biomechanics; 1995.
2. Gatev P, Thomas S, Kepple T, Hallett M. Feedforward ankle strategy of balance during quiet stance in adults. The Journal of physiology. 1999; 514(3):915–928. https://doi.org/10.1111/j.1469-7793.1999.915ad.x PMID: 9882761

3. Rocchi L, Chiari L, Horak F. Effects of deep brain stimulation and levodopa on postural sway in Parkinson's disease. Journal of Neurology, Neurosurgery & Psychiatry. 2002; 73(3):267–274. https://doi.org/10.1136/jnnp.73.3.267
4. Melzer I, Benjuya N, Kaplanski J. Postural stability in the elderly: a comparison between fallers and non-fallers. Age and ageing. 2004; 33(6):602–607. https://doi.org/10.1093/ageing/afh218 PMID: 15501837
5. Laessoe U, Voigt M. Anticipatory postural control strategies related to predictive perturbations. Gait & posture. 2008; 28(1):62–68. https://doi.org/10.1016/j.gaitpost.2007.10.001
6. Legrand A, Mazars KD, Lemoine C, Nougier V, Olivier I. Interference between oculomotor and postural tasks in 7–8-year-old children and adults. Experimental brain research. 2016; 234(6):1667–1677. https://doi.org/10.1007/s00221-016-4565-0 PMID: 26842856
7. Smalley A, White SC, Burkard R. The effect of augmented somatosensory feedback on standing postural sway. Gait & posture. 2017;.
8. Osu R, Franklin DW, Kato H, Gomi H, Domen K, Yoshioka T, et al. Short-and long-term changes in joint co-contraction associated with motor learning as revealed from surface EMG. Journal of neurophysiology. 2002; 88(2):991–1004. https://doi.org/10.1152/jn.2002.88.2.991 PMID: 12163548
9. Borg F, Finell M, Hakala I, Herrala M. Analyzing gastrocnemius EMG-activity and sway data from quiet and perturbed standing. Journal of Electromyography and Kinesiology. 2007; 17(5):622–634. https://doi.org/10.1016/j.jelekin.2006.06.004 PMID: 16890458
10. Ersal T, McCrory JL, Sienko KH. Theoretical and experimental indicators of falls during pregnancy as assessed by postural perturbations. Gait & posture. 2014; 39(1):218–223. https://doi.org/10.1016/j.gaitpost.2013.07.011
11. Horak FB, Nashner LM. Central programming of postural movements: adaptation to altered support-surface configurations. Journal of neurophysiology. 1986; 55(6):1369–1381. https://doi.org/10.1152/jn.1986.55.6.1369 PMID: 3734861
12. Carpenter MG, Frank JS, Silcher CP, Peysar GW. The influence of postural threat on the control of upright stance. Experimental brain research. 2001; 138(2):210–218. https://doi.org/10.1007/s002210100681 PMID: 11417462
13. Finley JM, Dhaher YY, Perreault EJ. Contributions of feed-forward and feedback strategies at the human ankle during control of unstable loads. Experimental brain research. 2012; 217(1):53–66. https://doi.org/10.1007/s00221-011-2972-9 PMID: 22169978
14. Doorenbosch CA, Harlaar J. A clinically applicable EMG–force model to quantify active stabilization of the knee after a lesion of the anterior cruciate ligament. Clinical Biomechanics. 2003; 18(2):142–149. PMID: 12550813
15. Vanderborght B, Van Ham R, Lefeber D, Sugar TG, Hollander KW. Comparison of mechanical design and energy consumption of adaptable, passive-compliant actuators. The International Journal of Robotics Research. 2009; 28(1):90–103. https://doi.org/10.1177/0278364908095333
16. Kim D. Compliant motion control for a compliant rehabilitation system. In: Rehabilitation Robotics (ICORR), 2015 IEEE International Conference on. IEEE; 2015. p. 422–427.
17. Horak FB, Henry SM, Shumway-Cook A. Postural perturbations: new insights for treatment of balance disorders. Physical therapy. 1997; 77(5):517–533. https://doi.org/10.1093/ptj/77.5.517 PMID: 9149762
18. Mulavara AP, Verstraete M, Simon B. Correlation between muscle activity and the center of pressure. In: Engineering in Medicine and Biology Society, 1994. Engineering Advances: New Opportunities for Biomedical Engineers. Proceedings of the 16th Annual International Conference of the IEEE. vol. 1. IEEE; 1994. p. 384–385.
19. Adkin AL, Frank JS, Carpenter MG, Peysar GW. Fear of falling modifies anticipatory postural control. Experimental brain research. 2002; 143(2):160–170. https://doi.org/10.1007/s00221-001-0974-8 PMID: 11880892
20. Siegmund GP, Sanderson DJ, Myers BS, Inglis JT. Rapid neck muscle adaptation alters the head kinematics of aware and unaware subjects undergoing multiple whiplash-like perturbations. Journal of biomechanics. 2003; 36(4):473–482. https://doi.org/10.1016/S0021-9290(02)00458-X PMID: 12600337
21. Schmid M, Bottaro A, Sozzi S, Schieppati M. Adaptation to continuous perturbation of balance: progressive reduction of postural muscle activity with invariant or increasing oscillations of the center of mass depending on perturbation frequency and vision conditions. Human movement science. 2011; 30(2):262–278. https://doi.org/10.1016/j.humov.2011.02.002 PMID: 21440318
22. Kennedy A, Bugnariu N, Guevel A, Sveistrup H. Adaptation of the feedforward postural response to repeated continuous postural perturbations. Neuroscience and Medicine. 2013; 4(01):45. https://doi.org/10.4236/nm.2013.41007

23. Schmid M, Sozzi S. Temporal features of postural adaptation strategy to prolonged and repeatable balance perturbation. Neuroscience letters. 2016; 628:110–115. https://doi.org/10.1016/j.neulet.2016.06.021 PMID: 27291456

24. Hirokawa S, Solomonow M, Luo Z, Lu Y, D'ambrosia R. Muscular co-contraction and control of knee stability. Journal of Electromyography and Kinesiology. 1991; 1(3):199–208. https://doi.org/10.1016/1050-6411(91)90035-4 PMID: 20870510

25. Milner TE, Cloutier C, Leger AB, Franklin DW. Inability to activate muscles maximally during cocontraction and the effect on joint stiffness. Experimental Brain Research. 1995; 107(2):293–305. https://doi.org/10.1007/BF00230049 PMID: 8773247

26. Blomqvist S, Wester A, Rehn B. Postural muscle responses and adaptations to backward platform perturbations in young people with and without intellectual disability. Gait & posture. 2014; 39(3):904–908. https://doi.org/10.1016/j.gaitpost.2013.11.018

27. Craig CE, Goble DJ, Doumas M. Proprioceptive acuity predicts muscle co-contraction of the tibialis anterior and gastrocnemius medialis in older adults' dynamic postural control. Neuroscience. 2016; 322:251–261. https://doi.org/10.1016/j.neuroscience.2016.02.036 PMID: 26905952

28. Winter DA, Patla AE, Prince F, Ishac M, Gielo-Perczak K. Stiffness control of balance in quiet standing. Journal of neurophysiology. 1998; 80(3):1211–1221. https://doi.org/10.1152/jn.1998.80.3.1211 PMID: 9744933

29. Morasso PG, Schieppati M. Can muscle stiffness alone stabilize upright standing? Journal of neurophysiology. 1999; 82(3):1622–1626. https://doi.org/10.1152/jn.1999.82.3.1622 PMID: 10482776

30. Warnica MJ, Weaver TB, Prentice SD, Laing AC. The influence of ankle muscle activation on postural sway during quiet stance. Gait & posture. 2014; 39(4):1115–1121. https://doi.org/10.1016/j.gaitpost.2014.01.019

31. Drillis R, Contini R, Bluestein M. Body segment parameters. New York University, School of Engineering and Science; 1969.

32. Masani K, Popovic MR, Nakazawa K, Kouzaki M, Nozaki D. Importance of body sway velocity information in controlling ankle extensor activities during quiet stance. Journal of Neurophysiology. 2003; 90(6):3774–3782. https://doi.org/10.1152/jn.00730.2002 PMID: 12944529

33. Lewek MD, Rudolph KS, Snyder-Mackler L. Control of frontal plane knee laxity during gait in patients with medial compartment knee osteoarthritis. Osteoarthritis and Cartilage. 2004; 12(9):745–751. https://doi.org/10.1016/j.joca.2004.05.005 PMID: 15325641

34. Iwamoto Y, Takahashi M, Shinkoda K. Differences of muscle co-contraction of the ankle joint between young and elderly adults during dynamic postural control at different speeds. Journal of physiological anthropology. 2017; 36(1):32. https://doi.org/10.1186/s40101-017-0149-3 PMID: 28764814

35. Scott SH. The computational and neural basis of voluntary motor control and planning. Trends in cognitive sciences. 2012; 16(11):541–549. https://doi.org/10.1016/j.tics.2012.09.008 PMID: 23031541

36. Cifrek M, Medved V, Tonković S, Ostojić S. Surface EMG based muscle fatigue evaluation in biomechanics. Clinical Biomechanics. 2009; 24(4):327–340. https://doi.org/10.1016/j.clinbiomech.2009.01.010 PMID: 19285766

37. Franklin DW, Osu R, Burdet E, Kawato M, Milner TE. Adaptation to stable and unstable dynamics achieved by combined impedance control and inverse dynamics model. Journal of neurophysiology. 2003; 90(5):3270–3282. https://doi.org/10.1152/jn.01112.2002 PMID: 14615432

38. Darainy M, Ostry DJ. Muscle cocontraction following dynamics learning. Experimental Brain Research. 2008; 190(2):153–163. https://doi.org/10.1007/s00221-008-1457-y PMID: 18584164

39. Datta A, Farmer S, Stephens J. Central nervous pathways underlying synchronization of human motor unit firing studied during voluntary contractions. The Journal of physiology. 1991; 432(1):401–425. https://doi.org/10.1113/jphysiol.1991.sp018391 PMID: 1886061

40. Cenciarini M, Loughlin PJ, Sparto PJ, Redfern MS. Medial-lateral postural control in older adults exhibits increased stiffness and damping. In: Engineering in Medicine and Biology Society, 2009. EMBC 2009. Annual International Conference of the IEEE. IEEE; 2009. p. 7006–7009.

41. Reynolds RF. The ability to voluntarily control sway reflects the difficulty of the standing task. Gait & posture. 2010; 31(1):78–81. https://doi.org/10.1016/j.gaitpost.2009.09.001

42. Nelson-Wong E, Appell R, McKay M, Nawaz H, Roth J, Sigler R, et al. Increased fall risk is associated with elevated co-contraction about the ankle during static balance challenges in older adults. European journal of applied physiology. 2012; 112(4):1379–1389. https://doi.org/10.1007/s00421-011-2094-x PMID: 21811766

43. Kearney RE, Stein RB, Parameswaran L. Identification of intrinsic and reflex contributions to human ankle stiffness dynamics. IEEE transactions on biomedical engineering. 1997; 44(6):493–504. https://doi.org/10.1109/10.581944 PMID: 9151483

44. Akazawa K, Milner TE, Stein RB. Modulation of reflex EMG and stiffness in response to stretch of human finger muscle. Journal of Neurophysiology. 1983; 49(1):16–27. https://doi.org/10.1152/jn.1983.49.1.16 PMID: 6827293

45. De Serres S, Milner T. Wrist muscle activation patterns and stiffness associated with stable and unstable mechanical loads. Experimental Brain Research. 1991; 86(2):451–458. https://doi.org/10.1007/BF00228972 PMID: 1756819

46. Trimble MH, Koceja DM. Effect of a reduced base of support in standing and balance training on the soleus H-reflex. International journal of neuroscience. 2001; 106(1-2):1–20. https://doi.org/10.3109/00207450109149734 PMID: 11264905

47. Taube W, Gruber M, Gollhofer A. Spinal and supraspinal adaptations associated with balance training and their functional relevance. Acta Physiologica. 2008; 193(2):101–116. https://doi.org/10.1111/j.1748-1716.2008.01850.x PMID: 18346210

48. Holl N, Zschorlich V. Neural control of joint stability during a ballistic force production task. Experimental brain research. 2011; 210(2):229–242. https://doi.org/10.1007/s00221-011-2618-y PMID: 21424843

49. Okada S, Hirakawa K, Takada Y, Kinoshita H. Relationship between fear of falling and balancing ability during abrupt deceleration in aged women having similar habitual physical activities. European journal of applied physiology. 2001; 85(6):501–506. https://doi.org/10.1007/s004210100437 PMID: 11718276

50. Young WR, Williams AM. How fear of falling can increase fall-risk in older adults: Applying psychological theory to practical observations. Gait & posture. 2015; 41(1):7–12. https://doi.org/10.1016/j.gaitpost.2014.09.006

51. Kuo AD, Zajac FE. A biomechanical analysis of muscle strength as a limiting factor in standing posture. Journal of Biomechanics. 1993; 26:137–150. https://doi.org/10.1016/0021-9290(93)90085-S PMID: 8505348

52. Desmurget M, Rossetti Y, Prablanc C, Jeannerod M, Stelmach GE. Representation of hand position prior to movement and motor variability. Canadian journal of physiology and pharmacology. 1995; 73(2):262–272. https://doi.org/10.1139/y95-037 PMID: 7621365

53. Wolpert DM, Ghahramani Z, Jordan MI. An internal model for sensorimotor integration. Science-AAAS-Weekly Paper Edition. 1995; 269(5232):1880–1882.

54. Bard C, Turrell Y, Fleury M, Teasdale N, Lamarre Y, Martin O. Deafferentation and pointing with visual double-step perturbations. Experimental brain research. 1999; 125(4):410–416. https://doi.org/10.1007/s002210050697 PMID: 10323286

55. Feldman AG, Levin MF. The origin and use of positional frames of reference in motor control. Behavioral and Brain Sciences. 1995; 18(04):723–744. https://doi.org/10.1017/S0140525X0004070X
